# Satiating Capacity of Plant-Based Meat in Realistic Meal Contexts at Home

**DOI:** 10.3390/foods12234280

**Published:** 2023-11-27

**Authors:** Elizabeth H. Zandstra, Ilse A. Polet, Gertrude G. Zeinstra, Anne J. Wanders, Garmt B. Dijksterhuis

**Affiliations:** 1 Unilever Foods Innovation Centre Wageningen, Bronland 14, 6708 WH Wageningen, The Netherlands; anne.wanders@unilever.com; 2 Human Nutrition & Health, Wageningen University and Research, Stippeneng 4, 6708 WE Wageningen, The Netherlands; 3 Wageningen Food and Biobased Research, Wageningen University and Research, Bornse Weilanden 9, 6708 WG Wageningen, The Netherlands; ilse.polet@wur.nl (I.A.P.); gertrude.zeinstra@wur.nl (G.G.Z.); g.b.dijksterhuis@uu.nl (G.B.D.); 4 Department Experimental Psychology, Utrecht University, Heidelberglaan 1, 3584 CS Utrecht, The Netherlands

**Keywords:** plant-based meat, animal meat, satiety, energy intake, compensation, liking, meal context, real-life

## Abstract

Plant-based meat substitutes replacing animal meat can potentially support the transition towards more sustainable diets. To enable the required transition, consumer acceptance of plant-based meat is essential. An important aspect of this is the feeling of satiety or being full after eating. This study determined the satiating capacity of both plant-based meat and animal meat in 60 adults under real-life in-home conditions. Participants consumed four fixed ready-to eat meals for lunch at home once per week. Two types of Indian curry with ‘chicken’ were investigated as well as two types of pasta Bolognese with ‘minced meat’. The two ‘chicken’ dishes and the two ‘minced meat’ dishes had the same recipe except for a gram-for-gram swap (125 g each) of either animal meat (chicken breast and minced meat) or plant-based (soy) meat. Results showed no difference in the satiating power of an animal meat dish and a plant-based meat dish when these were eaten as part of a full lunch meal at home. In addition, the meals did not result in energy nor macronutrient compensation during the rest of the day after consuming the meals. This occurred despite the caloric differences of the meals as a result of the real-life conditions (i.e., a lower energy content of the pasta with plant-based meat compared to the other meals). We conclude that meals with plant-based meat can be as satiating as meals with animal meat.

## 1. Introduction

Global animal meat consumption has increased tremendously in the past few decades [1]. The consumption and production of animal meat has major negative consequences on climate and the environment [2,3] and has been associated with negative health effects [4,5,6]. A shift to more sustainable plant-rich consumption patterns is therefore required to stop climate change and improve population health [7,8]. One way to support this transition is the use of plant-based meat substitutes, which are designed to mimic the appearance, taste, and texture of animal meat. Plant-based meat may be particularly suited to help current animal meat consumers and animal meat reducers (rather than vegetarians) to reduce their animal meat intake as they easily fit in people’s habitual animal meat-centred meals without further adjustment of their eating pattern [9,10]. Yet, while the market of plant-based meat is expanding globally, they are not widely accepted by consumers yet [11,12,13,14]. Consumer acceptance of plant-based meat is essential to enable the required transition [15,16,17]. An important aspect of this is the feeling of satiety or being full after eating [18]. Ideally, plant-based meat delivers optimal satiety, i.e., providing a satisfying experience that is not under- nor overfilling [19].

Satiety has been traditionally defined as the feeling of fullness and/or inhibition of hunger sensations after a meal resulting from the ingestion of food [20]. However, definitions of satiety have evolved over the years, leading to two functionally different terms, i.e., satiation and satiety [21]. The terms satiation and satiety are often confused and used interchangeably, although both describe sensations that are physiologically and behaviourally distinct [22]. Satiation relates to processes which terminate eating within a meal and determines the size of the meal and snack. Satiety is used to describe the post-ingestive processes that occur after eating a food or a meal has ended and inhibit further eating, which influences the frequency of meals and snacks. The two processes, therefore, control events going on within meals and between meals [23,24]. How satiation and satiety can be measured in humans has been the subject of a detailed review [25]. The present research focuses on the satiety of plant-based meat, which we quantified by tracking subjective feelings of hunger and fullness after eating dishes with plant-based meat and energy intake at the next meal or snack.

Little is known about the satiating capacity of plant-based meat. A study of Hoek et al. (2011) showed that people feel less full after eating plant-based products compared to animal meat products: ‘When I have vegetarian hamburgers, I always eat two’ [18]. The nutritional composition of food can affect satiety with protein exhibiting a more profound effect on satiety than fats and carbohydrates [26]. Meals with plant-based meat usually have a lower protein content than meals with animal meat, which may be the reason that people expected and perceived meals with plant-based meat to be less filling than meals with animal meat. That said, more recent research on the effect of plant-based meat on fullness and satiety showed more promising results. Research showed that people felt equally full after the consumption of meals with plant-based meat [27,28] or even more full [29,30] compared to equivalent meals prepared with animal meat. An explanation for this could be that product developers and nutritionists have been working hard to continuously improve the sensory attributes and nutritional quality of plant-based meat to make them the same or even better than animal meat, including the protein levels. However, due to the large variation in designs and food stimuli used amongst the studies available, the results should be interpreted cautiously and modestly. In addition, the studies offered isocaloric meals to the participants, which does not represent real-life situations of consumers preparing meals with plant-based meat. In everyday life, it is more likely that consumers replace the animal meat in a meal with (visually) the same amount of plant-based meat (one-to-one swap) without taking into account the difference in caloric value. Given the role of satiety in acceptance of plant-based meat, it is important to know what the effect is of a one-to-one plant-based meat swap for animal meat in a real-life context that is as similar as possible to how the product would usually be used by consumers.

The aim of this study was to investigate the satiating power of plant-based meat in a real-life meal context. The main research question was: How do feelings of satiety after consumption of a meal with plant-based (soy) meat differ from that of a meal with animal meat? Subjects consumed four fixed ready-to eat meals for lunch at home once per week. Two types of meals were investigated, Indian curry with ‘chicken’, and pasta Bolognese with ‘minced meat’. The animal and plant-based meals had exactly the same recipe except for a gram-for-gram swap (125 g each) of either animal meat or plant-based meat. The caloric content and macronutrient composition of the meals were not matched, since this is how consumers were expected to use meat substitutes in everyday life. This way, knowledge was gathered on the satiating capacity of plant-based meat in a natural consumption context.

## 2. Materials and Methods

### 2.1. Participants

In total, 68 participants were recruited for this study from the Wageningen University & Research participant database. Participants had to meet the following inclusion criteria: (1) aged between 18 and 65 years, (2) healthy (self-reported), (3) being an animal meat eater or flexitarian (i.e., excluding vegans, vegetarians, and pescatarians), (4) like Indian curry with chicken, and pasta Bolognese with minced meat (scoring ≥ 4 on a 7-point scale). Participants were not informed about the actual purpose of the study (i.e., assessing satiating power of plant-based meat) but were informed that the study consisted of tasting and evaluating different meals during lunch at home. Participants signed informed consent prior to the start of the experiment and received €50 on study completion. Seven participants withdrew from participation just before the start of the study, and one participant was excluded because of dropping out halfway the study due to personal reasons: their data were excluded from statistical analysis, leading to a final sample size of 60 participants. The study was evaluated by the METC Brabant as not being subject to the WMO Medical Research Involving Human Subjects Act (NW2021-51). Ethical approval was granted by the Social Sciences Ethics Committee of Wageningen University, indicating that it complied with the Netherlands Code of Conduct for Research Integrity.

### 2.2. Products

This study used minced beef meat and chicken breast that was purchased from the local butcher and plant-based minced meat and plant-based chicken that was commercially available in the supermarket (brand: The Vegetarian Butcher; soy-based meat with taste, texture and appearance mimicking animal meat). Two types of meals were investigated with exactly the same recipe except for a gram-for-gram swap (125 g each) of either meat or soy-based meat: (1) pasta Bolognese with minced beef meat (Pasta Animal Meat), (2) pasta Bolognese with plant-based minced meat (Pasta Plant-Based Meat), (3) curry tandoori with spinach, rice and chicken breast (Curry Animal Meat), and (4) curry tandoori with spinach, rice and plant-based chicken (Curry Plant-Based Meat). All ingredients used in the pasta and curry dish were commercially available (pasta fusilli, sunflower oil, mushroom, carrot, onion, zucchini, green bell pepper, tomato, vegetable bouillon powder, Italian herbs, rice, spinach, garlic, ginger, coconut milk). The total caloric value of the pasta meals was 637 kJ/100 g for the Pasta Animal Meat and 367 kJ/100 g for the Pasta Plant-Based Meat, and the total caloric value of the curry meals was 533 kJ/100 g for the Curry Animal Meat and 552 kJ/100 g for the Curry Plant-Based Meat. Table 1 gives an overview of the energy and nutritional composition of the four meals.

We did not correct for differences in energy and macronutrients of the test meals as we wanted to test real-life conditions of how consumers use plant-based meat in their meals at home (one-to-one swap). As plant-based meat is sold as a ready-to-eat and seasoned product, we did correct for differences in seasoning and taste between the animal meat and plant-based meat condition by adding extra vegetable bouillon powder to the meal with animal meat (1.9 g for the curry dish and 3.5 g for the pasta dish). The total weight of each of the meals was 400–402 g. The weight of the meals was kept equal to all participants with no differentiation for sex or body weight classes. The recipes for the meals with the complete list of ingredients are available on request.

### 2.3. Procedure

We collected the data in July 2021, which was a time period with lockdown measures in The Netherlands because of the COVID-19 pandemic. Participants ate the test meals during lunch at home. The meals were prepared at the kitchen facilities of the university and were kept refrigerated at 4 °C. Participants picked up the meals the day before each test day. On test days, participants were instructed to eat their usual breakfast at 8:00 h at home. They were asked to eat the same breakfast (that they could choose themselves) on each of the test days and to keep activity and stress levels similar on each day before the test day and on each test day itself. Between breakfast and lunch, participants were asked to drink only water, coffee or tea without milk and sugar. At 11:55 h, participants heated the test meal in their microwave and started eating the meal at 12:00 h at home. Participants completed an online questionnaire on subjective feelings of hunger and satiety just before consumption of the meal and at 15, 45, 75, 105 and 150 min after that. Participants also rated liking (at first bite and after finishing the full meal) and taste perception of the meal.

**Figure 1:**
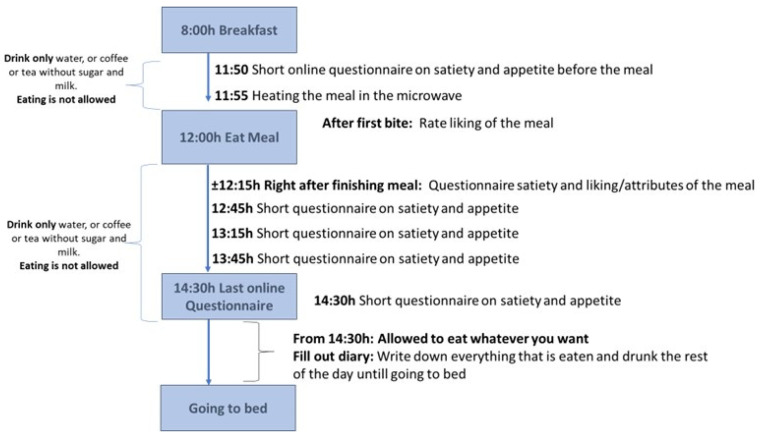
Schematic overview of test days.

Participants used their own mobile devices to answer all online questionnaires. They received a text message on their mobile device at the appropriate moments to remind them of heating the test meal and filling out the questionnaires. Each text message contained the link to the questionnaire. If participants did not fill in the questionnaire in time, they were called to remind them. After finishing all the satiety questionnaires (at 14:30 h, 150 min after starting the meal), participants were asked to write down what they ate and drank during the rest of that day in a small paper diary that they received from the research team. Figure 1 shows a schematic overview of the test days. At the end of the four sessions, participants completed (online) questionnaires on meat attachment, health and environmental orientation in food choices, and attitudes towards animals.

### 2.4. Measurements

#### 2.4.1. Appetite, Liking and Taste Perception

Participants rated their appetite just before starting the lunch meal, immediately after finishing the meal (15), and 45, 75, 105, and 150 min after starting the meal. Appetite was assessed using six items that were scored on a 100 mm visual analogue scale ranging from ‘not at all’ to ‘very much’ for ‘Feeling full’, ‘Hungry’, ‘Appetite for a meal’, ‘Appetite for a snack’, ‘Appetite for something sweet’ and ‘Appetite for something savoury’. In addition, just before consumption of each meal, participants rated their desire to eat that specific meal. After one bite, participants rated their liking of the meal. After finishing the meal, they rated their liking of the full meal as well their separate liking of the sauce and of the (animal/plant-based) meat. Right after finishing the meal, participants also rated the meal on perceived sweetness, saltiness, bitterness, sourness, juiciness, firmness of the (animal/plant-based) meat, and creaminess of the sauce. Desire to eat, liking, and the sensory attributes were all scored on a 7-point scale, ranging from ‘not at all’ to ‘very much’.

#### 2.4.2. Energy Compensation during the Afternoon and Evening

The participants kept a record of what they ate or drank during the afternoon and evening (from 14:30 h until going to bed; see also Figure 1) on each of the four experimental days, using a pen and paper diary. This information was used to measure energy and macronutrient intake during the rest of the day (after finishing the last satiety questionnaire at 14:30 h). Participants received detailed instructions for completing the diaries before the start of the study. These instructions were included in each diary as well. The diaries were checked and coded by experienced dieticians. In case of unclear food records, participants were asked about this the next time they picked up their meal (within a week).

#### 2.4.3. Psychosocial Variables Related to Meat Consumption

After the final test day, participants completed different questionnaires on their willingness to try novel foods (food neophobia), food choice motives and attitudes towards animal meat consumption. Completion of this questionnaire set took about 15 min. The Food Neophobia Scale [31] is a 10-item questionnaire that measures a reluctance to eat and/or avoid novel foods. Responses were given on a 7-point Likert scale ranging from ‘strongly disagree’ to ‘strongly agree’. Four dimensions of the Food Choice Motives questionnaire were included to measure the motives that consumers find important in their daily food choices in relation to health, weight control, natural content, environment, and animals [32,33]. Each dimension consisted of 3 items that were scored on a 7-point scale ranging from ‘very unimportant’ to ‘very important’. Interest in reducing animal meat consumption (i.e., what behavioural stage of change participants were in) was measured with one question with five answer options, which were based on the five stages of the Trans Theoretical Model of Behavioural Change [34]. Participants were then regrouped into three ‘Interest in Reducing Animal Meat’ groups, i.e., ‘not aware, no action’ group (stage 1), ‘aware, no action’ group (stages 2 and 3) and ‘aware and action’ group (stages 4 and 5). Attitudes towards limiting animal meat consumption were collected via three questions on ‘Importance’, ‘Healthiness’, and ‘Good for the environment’. Responses were rated on a 7-point scale ranging from ‘not at all’ to ‘very’. To assess animal meat consumption frequency, participants were asked to indicate on how many days per week they consume a main meal that contains animal meat: 1–3 (infrequent animal meat consumers), 4–5 (moderate animal meat consumers), and 6–7 (frequent animal meat consumers). Since no vegans or vegetarians participated in the study, the “never” option was not given. Animal meat attachment was measured using the 16-item Meat Attachment Scale of Graça et al. (2015) on hedonism (4 items), affinity (4 items), entitlement (3 items), and dependence (5 items) [35]. Responses were on a 7-point Likert scale ranging from “strongly disagree” to “strongly agree”.

### 2.5. Statistical Analysis

Data are presented as means and standard errors. Statistical analyses were carried out with the statistical software package SPSS version 28.0.1.1 [36]. We considered differences significant at *p* < 0.05. A three-factor repeated measures ANOVA was carried out separately for each subjective feeling of hunger and satiety with the six time points after the meal (T0, T15, T45, T75, T105, T150), the two meal types (Pasta, Curry) and the two meat types (Meat, Plant-Based) as factors. In both repeated measures ANOVA analyses of the ‘Feeling full’ and ‘Hungry’ scores, Mauchly’s test showed a significant deviation of Sphericity, so the Greenhouse-Geisser and Huynh-Feldt corrected *F*-statistics (of which we only report the former here) were used to assess the significance of the factors. A two-factor (Meal type × Meat type) repeated measures ANOVA was performed on the liking and sensory data. F-values and Bonferroni corrected *p*-levels are reported. Energy compensation was calculated based on the reported consumption from T150 (150 min after starting the meal) until going to bed by calculating consumed (total) energy, protein, fat, carbohydrates, fibre, and alcohol (all in kJ). The main interest was any difference in compensatory eating behaviour between the animal meat and plant-based meat meals (Pasta, Curry).

## 3. Results

### 3.1. Participants

Table 2 shows the main characteristics of the 60 participants who completed the study. Most participants indicated to be in the ‘aware and action’ stage (70%) in terms of Interest in Reducing Meat. Participants scored relatively low on food neophobia (22.6 ± 7.0 on a scale from 10 to 70) and had a slightly positive attitude towards cutting down on meat consumption (5.1 ± 1.1 on a 7-point scale). In addition, they scored relatively high on meat attachment (subscales Dependence, Entitlement, Hedonic and Affinity ranging from 3.1 to 5.9 on 7-point scales).

### 3.2. Subjective Feelings of Appetite

For subjective feelings of appetite, only the means of the groups for all analyses are reported, as there were no statistically significant differences by sex, age, food neophobia, meat eating frequency or meat attachment across time moments after the meal (*p* > 0.05). For subjective feelings of hunger, however, there was a significant difference for sex (F = 21.6, *p* < 0.05), with men having overall higher scores than women (30.2 ± 1.2 and 23.4 ± 0.8, respectively), but not for meal (F = 0.96, *p* = 0.41) nor for their interaction (F = 0.08, *p* = 0.97).

**Figure 2:**
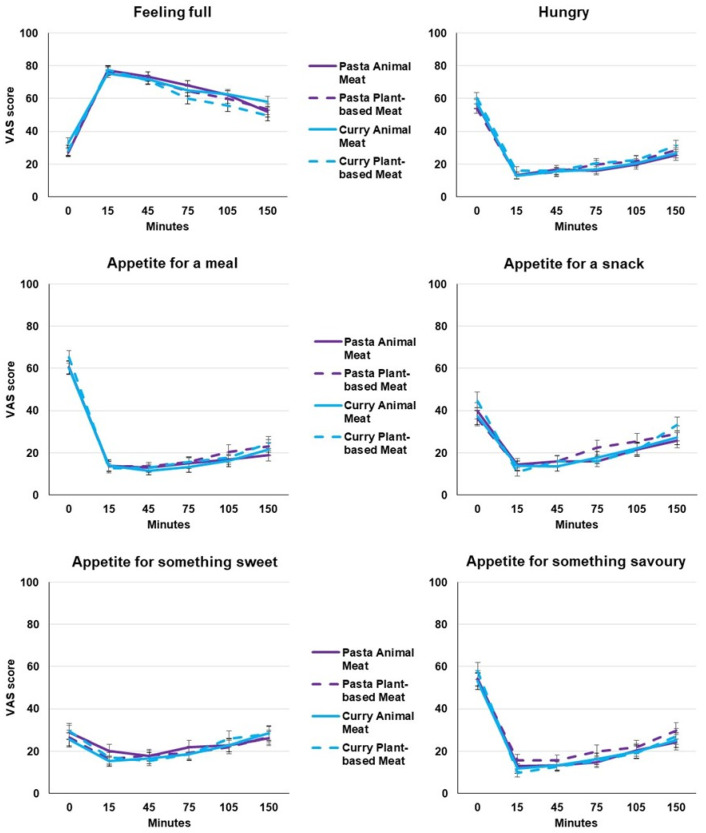
Mean satiety ratings ± SE from 0 (weak) to 100 (strong) after the different meals with animal meat and plant-based meat (n = 60). Appetite ratings were rated just before starting the meal (0 min), immediately after finishing the meal (15 min), and 45, 75, 105, and 150 min after starting the meal.

Figure 2 shows the absolute appetite ratings of the items ‘Feeling full’, ‘Hungry’, ‘Appetite for a meal’, ‘Appetite for a snack’, ‘Appetite for something sweet’ and ‘Appetite for something savoury’. The mean responses for the items ‘Hungry’ and ‘Appetite for a meal’ were similar to the responses of the items ‘Appetite for a snack’, ‘Appetite for something sweet’ and ‘Appetite for something savoury’, while the curve of responses of the item ‘Feeling full’ was the reverse. Analysis of the appetite ratings revealed a significant effect across the different time points for the meals (all *p* < 0.001). For example, the T0 time points (just before the meal) were significantly different from almost all the other post-meal time points, which was as expected. The only clear deviation from this pattern is seen for ‘Appetite for something sweet’, which had much less significant differences between the time points (not tabulated, but see the much more flat curves in Figure 2).

For ‘Feeling full’, the only significant main effect was time after meal (Table 3), where all the time points differed significantly from each other (pairwise differences *p* < 0.001 thru *p* = 0.048, Bonferroni corrected, not tabulated). The ‘Hungry’ scores showed a similar picture (see Table 3), but here, the first and the last time point are significantly different from all other time points (*p* < 0.001), and the other time points do not differ (pairwise differences 0.098 < *p* < 1.00, Bonferroni corrected, not tabulated).

The mean appetite ratings (‘Feeling full’, ‘Hungry’, ‘Appetite for a meal’, ‘Appetite for a snack’, ‘Appetite for something sweet’, ‘Appetite for something savoury’) did not show significant effects of either Meal type (Pasta, Curry) and Meat type (Meat, Plant-Based) (0.09 < *p* < 0.98, Table 3).

In addition, there was no significant difference between the energy and macronutrient intake during the rest of the day, with an energy intake of 5.0 ± 0.3 MJ, 4.8 ± 0.3 MJ, 4.8 ± 0.3 MJ and 5.1 ± 0.3 MJ after consuming Pasta Animal Meat, Pasta Plant-Based Meat, Curry Animal Meat and Curry Plant-Based Meat, respectively (all *p* values > 0.05). Interestingly, this occurred despite the caloric differences between the different meals.

### 3.3. Liking and Taste Perception

Table 4 shows the means ± SE for desire-to-eat, liking and sensory attribute scores. Overall, the meals were moderately liked with ratings ranging from 3.2 to 5.5 on 7-point scales. The pattern of results obtained for liking for the full meal were similar to the pattern of results for liking at first bite and the liking of the sauce. Overall, participants liked the curry meals better than the pasta meals, and they like the meals with meat better than the plant-based meals (Table 4). The animal meat was significantly preferred over the plant-based meat, and the curry meal was perceived as being sweeter, saltier as well as more sour than the pasta meal (Table 4). The only effect of bitter taste was seen in the plant-based meat being perceived as more bitter than the animal meat (Table 4).

Table 5 shows a summary of the outcomes of the repeated measures ANOVAs for desire to eat, liking and sensory attribute scores.

The desire-to-eat ratings did not show an effect of Meal type, Meat type nor their interaction (Table 5). The liking ratings (1st bite, full meal, sauce) show significant main effects of Meal type and Meat type and no interactions (Table 5).

## 4. Discussion

The results of this study indicate that there is no difference in the satiating power of an animal meat dish and a plant-based meat dish when these were eaten as part of a full meal at lunch at home. The scores on fullness, hunger, and appetite as well as food intake on the remainder of the day were not affected by having consumed animal meat or plant-based meat.

The mean appetite and satiety ratings for meals with plant-based meat were similar to the meals with animal meat, and there was no significant difference between the meals in energy compensation during the rest of the day after consuming the meals. These findings are consistent with the studies of Nielsen et al. (2018) [27] and Williamson et al. (2006) [28] that showed that people felt equally full after the consumption of meals with plant-based meat compared to equivalent meals prepared with animal meat. However, our results contradict other research that even showed higher scores on satiety or satiation [29,30] or the opposite: that people expected to be less full of meals with plant-based meat [18]. These different results could be attributed to differences in methodologies used, i.e., actual eating of meals [our study]; [27,28,30] vs. asking via a survey [18], fixed portions of meals [our study]; [27,28] vs. ad libitum meals where people consumed the meals until satiated [30] and different ingredients used, i.e., soy [our study]; [30] vs. tofu [28,29] vs. mycoprotein [28] vs. fava beans/split peas [27]. Also, in most of these studies, people were offered iso-caloric meals, whereas in our study, we did not match the caloric content of the meals but used a gram-for-gram swap of animal meat with plant-based meat to better reflect how consumers use plant-based meat in everyday life.

Interestingly, despite the caloric and macronutrient differences between the different meals, we found no difference in the satiating power of the meals when eaten as part of a full meal at lunch at home. Research suggests three possibilities that could explain this finding. First, the fibre content of the meals with plant-based meat was higher than the meals with animal meat, and the protein content of the plant-based meals was lower than the meals with animal meat. Fibre and protein have been shown to enhance satiety and reduce food intake [37,38] and it could well be that the higher fibre content of plant-based meat may have compensated for the lower protein content especially when eaten as part of a full meal. Secondly, based on the energy content, a difference in satiating power would have been expected, especially for the pasta dish with plant-based (minced) meat which had a lower energy content compared to the other meals (1469 kJ versus 2132–2548 kJ). That said, research has consistently shown that high-fat, energy-dense foods (‘hidden fat’) facilitate passive overconsumption, as these foods do not provide sensory signals in accordance with the actual nutrient content [39]. The fat content of the animal (minced) meat was higher than the fat content of the plant-based meat (respectively, 30.5 vs. 2.1 g per serving), which could have been the reason for the unexpected findings on the satiating power of the meals. Thirdly, the effects of ingestion of a particular food product on satiety depend not only on the energy and macronutrient composition of a food but also on beliefs and expectations, sensory factors (taste and texture), consumer’s familiarity with that product and their individual sensitivity to satiety [25,40,41]. Social influences can impact satiation as well [42], and all of these factors interact to modify overall appetite in a holistic product experience [25]. An increased understanding of these complex interactions will bring innovative directions for the development of plant-based products with optimal satiety, which consumers will be able to enjoy over and over again [43].

People eat more of foods they like more [44,45]. The meals with animal meat in the present study were slightly better liked than the meals with plant-based meat. This is in line with previous research [15,46] and reinforces the fact that it is important to continuously improve the sensory profile (i.e., taste and texture) of plant-based meat. The lower liking scores of plant-based meat could also be a motivational barrier for consumers to switch to plant-based meat. Previous research has shown that motivational barriers that withhold consumers from switching to plant-based meat include the following: a lower sensory appeal of plant-based compared to animal meat, unfamiliarity with plant-based meat, social–cultural aspects, a tendency to avoid new foods, and negative attitudes and beliefs about the sensory appeal of plant-based meat (e.g., meat attachment) [12,18,47,48]. The difference in liking may have impacted feelings of satiety as well despite no differences in satiating power between the animal meat dishes and the plant-based dishes in this study. So far, research on the impact of liking on satiety has shown inconsistent results. Studies showed that an increased liking resulted in either increased feelings of satiety or satiation [49,50,51,52], decreased feelings of satiety or satiation [53,54], or no influence on satiety or satiation [45,55]. The study of Zandstra et al. (2000) was the only study that investigated longer-term effects of pleasantness of a food on satiety over repeated exposure [52]. This study found that the pleasantness of a less preferred food remained unaltered, while the satiety and actual intake of that food increased over time. On this basis, we would encourage investigating the effects of repeated consumption of meals with plant-based meat on satiety and intake over a longer time in future studies, i.e., over weeks or months. In addition, recent research showed that children prefer plant-based meat to resemble animal meat in appearance, taste and texture, as with adults [56]. For future studies, it would be interesting to investigate the impact of eating a dish with plant-based meat on satiety in different age groups such as children, young adults, and the elderly [57].

Real-life consumption behaviour does not take place in isolation; rather, it happens in a specific physical (e.g., dining room) and social (e.g., family) context [58]. For the present study, it was a deliberate choice to elicit satiety ratings of plant-based meat in a natural consumption context at home rather than eliciting them under controlled laboratory conditions. We wanted the experience and evaluation of plant-based meat to be in a context that would be as similar as possible to how the product would usually be used by consumers [59]. Depending on the research question itself, there is always a trade-off to make between precision and accuracy on the one side and external validity on the other [40]. Satiety data gathered under laboratory conditions are usually accurately related to eating behaviour, but the circumstances are not natural (because people do not normally eat alone in a sensory booth isolated from social stimuli). On the other hand, eating under free-living conditions can be considered natural but is often not precisely related to the eating behaviour that forms the target of interest [40]. A recent study from De Wijk et al. (2019) investigated the effect of test location (lab vs. home) on the eating behaviour of plant-based meat and showed significant changes, albeit small, in eating behaviour between home and lab testing conditions [58]. The test foods consumed at home were eaten at a faster rate with greater chewing intensity than the same foods consumed in the laboratory. It is well known that eating rate can influence satiety, with faster eating rates being associated with reduced satiety [60]. For the present study, it was therefore also more relevant to assess the plant-based meat in a realistic context in order to increase the external validity of the results [61].

There are some limitations of the study that should be mentioned. The results are based on plant-based meat made with soy in two dishes. It is recommended to replicate the study for other plant-based alternatives to animal meat, e.g., based on pulses, mycoprotein or algae, and for a broader range of dishes with more variation in energy and macronutrient composition to substantiate the findings. In addition, our population was skewed towards women and participants from Wageningen and surroundings who may have been more aware and open to the required protein transition than the general population, which might limit generalisability to a wider population. However, although 70% of the participants indicated they started reducing their animal meat consumption six months or longer ago, the majority (65%) of the participants indicated that they eat animal meat at least 4 days per week. Finally, subjects were instructed to complete food diaries, and on return to the laboratory, the research team reviewed the records in the presence of the subject. Absolute honesty and accuracy cannot be guaranteed in this type of measurement. This method might also lack the sensitivity to detect small energy adjustments given the relatively large standard deviations in estimated energy intake. As discussed before, this diary method is clearly a compromise between the restrictions in eating behaviour of laboratory studies and the spontaneous behaviour in free-living conditions [62].

## 5. Conclusions

To conclude, this study showed no difference in the satiating power of animal meat and plant-based meat when these were eaten as part of a full meal at lunch at home. The higher fibre content of plant-based meat may have compensated for the lower protein content. While these results are promising, future research is required to establish what happens over repeated in-home consumption of a range of plant-based meats over longer periods of time. Our study findings suggest that a gram-by-gram swap of plant-based meat might be a useful instrument in reducing animal meat consumption, since this strategy addresses the core consumer determinants of preference, satiety, and convenience.

## Figures and Tables

**Table 1:** Energy and nutritional composition of meals (per serving of 400 g).

Meals	Total Energy (kJ)	Protein (g)	Fat (g)	Fat saturated (g)	Carbohydrates (g)	Fibre (g)	Sodium (mg)
Pasta Animal Meat	2548	46.5	30.5	8.5	35.2	4.2	1591
Pasta Plant-Based Meat	1469	38.2	2.1	0.4	70.9	12.2	1523
Curry Animal Meat	2132	42.3	19.5	5.3	39.3	2.5	1854
Curry Plant-Based Meat	2209	31.5	22.1	5.0	44.9	12.0	1832

**Table 2:** Characteristics of the participants and psychosocial variables related to animal meat consumption.

	Mean (SE)
Sex (*m*/*w*) (*n*)	20/40
Age (years)	48.0 ± 13.7
Frequency of animal meat consumption (%)	
Infrequent (1–3 days a week)	35.0
Moderate (4–5 days a week)	56.7
Frequent (6–7 days a week)	8.3
Interest in Reducing Animal Meat (%) ^a^	
Not aware, no action	16.7
Aware, no action	13.3
Aware and action	70.0
Food neophobia ^b^	22.6 (6.9)
Attitude towards animal meat reduction ^c^	5.1 (1.1)
Food Choice Motives ^c^	
Health	5.6 (0.9)
Animal	5.1 (1.3)
Weight Control	4.3 (1.4)
Natural Content	4.3 (1.5)
Environment	4.4 (1.5)
Animal Meat Attachment ^d^	
Affinity	5.8 (1.0)
Hedonic	4.3 (1.4)
Entitlement	3.5 (1.4)
Dependence	3.1 (1.4)

^a^ The three Interest in Reducing Animal Meat groups were based on the five stages of the Trans Theoretical Model of Behavioural Change [34], i.e., ‘not aware, no action’ group (stage 1), ‘aware, no action’ group (stages 2 and 3) and ‘aware and action’ group (stages 4 and 5). ^b^ Answers were measured on a 7-point scale, total sum scale ranges from 10 to 70 (low to high). ^c^ Answers were measured on a 7-point importance scale (negative to positive). ^d^ Answers were measured on a 7-point scale (low to high).

**Table 3:** Overview F-values (Greenhouse-Geisser corrected) and *p*-levels for the three-factor (Time points (6) × Meal type (2) × Meat type (2)) repeated measures ANOVAs (significant levels, *p* < 0.05 in bold).

Feelings of Appetite	Time after Meal		Meal Type		Meat Type		Interactions
	F-Value	*p*	F-Value	*p*	F-Value	*p*	
Feeling full	**87.92**	**<0.001**	0.14	0.71	2.99	0.09	None significant
Hungry	**95.09**	**<0.001**	1.79	0.19	2.23	0.14	None significant
For a meal	**118.20**	**<0.001**	0.08	0.78	2.45	0.12	None significant
For a snack	**28.45**	**<0.001**	0.01	0.91	0.93	0.34	None significant
For sweet	**6.63**	**<0.001**	0.002	0.97	0.001	0.98	None significant
For savoury	**80.82**	**<0.001**	0.56	0.46	1.25	0.27	None significant

**Table 4:** Mean desire to eat, liking and sensory attribute intensity scores (±SE) for Meal types (Curry and Pasta) and Meat types (Animal and Plant-Based) rated on 7-point scales. Significantly higher scores are in bold, non-significantly differing means in italic against grey.

	Meal		Meat	
	Curry	Pasta	Animal	Plant-Based
Desire to eat	*5.2 (0.2)*	*5.1 (0.1)*	*5.2 (0.2)*	*5.1 (0.1)*
Liking at 1st bite	**5.2** (0.1)	4.6 (0.1)	**5.2** (0.1)	4.6 (0.2)
Liking full meal	**5.0** (0.1)	4.3 (0.2)	**5.1** (0.1)	4.2 (0.1)
Liking sauce	**5.1** (0.1)	3.7 (0.2)	**4.8** (0.2)	4.0 (0.1)
Liking meat	*4.3 (0.2)*	*4.0 (0.2)*	**4.8** (0.2)	3.5 (0.2)
Sweet	**2.9** (0.2)	2.5 (0.1)	*2.8 (0.2)*	*2.7 (0.2)*
Salty	**4.1** (0.1)	3.5 (0.2)	*3.7 (0.2)*	*3.8 (0.2)*
Bitter	*1.9 (0.1)*	*1.9 (0.1)*	1.8 (0.1)	**2.0** (0.1)
Sour	**2.4** (0.2)	2.1 (0.1)	*2.2 (0.2)*	*2.2 (0.1)*
Juicy	**4.3** (0.1)	2.7 (0.1)	**3.7** (0.1)	3.2 (0.1)
Firm	**5.2** (0.1)	4.8 (0.2)	*5.1 (0.1)*	*4.9 (0.2)*
Creamy	**3.2** (0.2)	1.9 (0.1)	**2.8** (0.2)	2.4 (0.1)

**Table 5:** Overview F-values and *p*-levels two factor (Meal type (2) × Meat type (2)) repeated measures ANOVAs (Bonferroni-corrected significant levels, *p* < 0.05 in bold).

	Meal Type		Meat Type		Interaction	
	F-Value	*p*	F-Value	*p*	F-Value	*p*
Desire to eat	0.36	0.55	1.45	0.23	0.03	0.86
Liking at 1st bite	**23.26**	**<0.001**	**13.0**	**<0.001**	0.81	0.37
Liking full meal	**13.90**	**<0.001**	**38.3**	**<0.001**	0.67	0.42
Liking sauce	**64.10**	**<0.001**	**23.4**	**<0.001**	3.29	0.08
Liking meat	2.71	0.11	**53.7**	**<0.001**	1.52	0.22
Sweet	**6.63**	**0.013**	0.20	0.66	0.08	0.78
Salty	**22.80**	**<0.001**	0.35	0.56	0.57	0.45
Bitter	0.26	0.61	**4.85**	**0.03**	0.17	0.68
Sour	**4.44**	**0.04**	0.14	0.71	0.62	0.44
Juicy	**114.10**	**<0.001**	**9.09**	**0.004**	**14.90**	**<0.001**
Firm	**6.51**	**0.01**	1.10	0.30	0.02	0.89
Creamy	**87.0**	**<0.001**	**6.92**	**0.01**	3.34	0.07

## Data Availability

The data that support the findings of this study are available on request from the corresponding author. The data are not publicly available due to it involves human research data, we are open to share it, but in close consultation with the persons who request it and are going to use it. This is in agreement with the METC protocol.

## References

[1] Parlasca M.C., Qaim M. (2022). Meat Consumption and Sustainability. Annu. Rev. Resour. Econ..

[2] Godfray H.C.J., Aveyard P., Garnett T., Hall J.W., Key T.J., Lorimer J., Pierrehumbert R.T., Scarborough P., Springmann M., Jebb S.A. (2018). Meat Consumption, Health, and the Environment. Science.

[3] Springmann M., Clark M., Mason-D’Croz D., Wiebe K., Bodirsky B.L., Lassaletta L., de Vries W., Vermeulen S.J., Herrero M., Carlson K.M. (2018). Options for Keeping the Food System within Environmental Limits. Nature.

[4] Ekmekcioglu C., Wallner P., Kundi M., Weisz U., Haas W., Hutter H.P. (2018). Red Meat, Diseases, and Healthy Alternatives: A Critical Review. Crit. Rev. Food Sci. Nutr..

[5] Wang X., Lin X., Ouyang Y.Y., Liu J., Zhao G., Pan A., Hu F.B. (2016). Red and Processed Meat Consumption and Mortality: Dose-Response Meta-Analysis of Prospective Cohort Studies. Public Health Nutr..

[6] Yip C.S.C., Lam W., Fielding R. (2018). A Summary of Meat Intakes and Health Burdens. Eur. J. Clin. Nutr..

[7] WHO-FAO (2019). Sustainable Healthy Diets—Guiding Principles.

[8] Willett W., Rockström J., Loken B., Springmann M., Lang T., Vermeulen S., Garnett T., Tilman D., DeClerck F., Wood A. (2019). Food in the Anthropocene: The EAT–Lancet Commission on Healthy Diets from Sustainable Food Systems. Lancet.

[9] Caputo V., Sogari G., Van Loo E.J. (2023). Do Plant-Based and Blend Meat Alternatives Taste like Meat? A Combined Sensory and Choice Experiment Study. Appl. Econ. Perspect. Policy.

[10] Fiorentini M., Kinchla A.J., Nolden A.A. (2020). Role of Sensory Evaluation in Consumer Acceptance of Plant-Based Meat Analogs and Meat Extenders: A Scoping Review. Foods.

[11] Boukid F. (2021). Plant-Based Meat Analogues: From Niche to Mainstream. Eur. Food Res. Technol..

[12] Jahn S., Furchheim P., Strässner A.M. (2021). Plant-Based Meat Alternatives: Motivational Adoption Barriers and Solutions. Sustainability.

[13] Michel F., Hartmann C., Siegrist M. (2021). Consumers’ Associations, Perceptions and Acceptance of Meat and Plant-Based Meat Alternatives. Food Qual. Prefer..

[14] Onwezen M.C., Bouwman E.P., Reinders M.J., Dagevos H. (2021). A Systematic Review on Consumer Acceptance of Alternative Proteins: Pulses, Algae, Insects, Plant-Based Meat Alternatives, and Cultured Meat. Appetite.

[15] Spendrup S., Hovmalm H.P. (2022). Consumer Attitudes and Beliefs towards Plant-Based Food in Different Degrees of Processing—The Case of Sweden. Food Qual. Prefer..

[16] Zandstra E.H., Weegels M.F., Van Spronsen A.A., Klerk M. (2004). Scoring or Boring? Predicting Boredom through Repeated in-Home Consumption. Food Qual. Prefer..

[17] Zandstra E.H. (2018). Food Reward Matters.

[18] Hoek A.C., Luning P.A., Weijzen P., Engels W., Kok F.J., de Graaf C. (2011). Replacement of Meat by Meat Substitutes. A Survey on Person- and Product-Related Factors in Consumer Acceptance. Appetite.

[19] Chambers L., McCrickerd K., Yeomans M.R. (2015). Optimising Foods for Satiety. Trends Food Sci. Technol..

[20] Blundell J. (1991). Pharmacological Approaches to Appetite Suppression. Trends Pharmacol. Sci..

[21] Yeomans M.R., Meiselman H.L. (2019). Satiety. Handbook of Eating and Drinking: Interdisciplinary Perspectives.

[22] Blundell J., De Graaf C., Hulshof T., Jebb S., Livingstone B., Lluch A., Mela D., Salah S., Schuring E., Van Der Knaap H. (2010). Appetite Control: Methodological Aspects of the Evaluation of Foods. Obes. Rev..

[23] Blundell J.E., Halford J.C.G. (1994). Regulation of Nutrient Supply: The Brain and Appetite Control. Proc. Nutr. Soc..

[24] Blundell J.E., Green S., Burley V. (1994). Carbohydrates and Human Appetite. Am. J. Clin. Nutr..

[25] Forde C.G., Ares G., Varela P. (2018). Chapter 7—Measuring Satiation and Satiety. Methods in Consumer Research.

[26] Rolls B.J., Hetherington M., Burley V.J. (1988). The Specificity of Satiety: The Influence of Foods of Different Macronutrient Content on the Development of Satiety. Physiol. Behav..

[27] Nielsen L.V., Kristensen M.D., Klingenberg L., Ritz C., Belza A., Astrup A., Raben A. (2018). Protein from Meat or Vegetable Sources in Meals Matched for Fiber Content Has Similar Effects on Subjective Appetite Sensations and Energy Intake—A Randomized Acute Cross-over Meal Test Study. Nutrients.

[28] Williamson D.A., Geiselman P.J., Lovejoy J., Greenway F., Volaufova J., Martin C.K., Arnett C., Ortego L. (2006). Effects of Consuming Mycoprotein, Tofu or Chicken upon Subsequent Eating Behaviour, Hunger and Safety. Appetite.

[29] Klementova M., Thieme L., Haluzik M., Pavlovicova R., Hill M., Pelikanova T., Kahleova H. (2019). A Plant-Based Meal Increases Gastrointestinal Hormones and Satiety More than an Energy-and Macronutrient-Matched Processed-Meat Meal in T2d, Obese, and Healthy Men: A Three-Group Randomized Crossover Study. Nutrients.

[30] Muhlhausler B.S., Belobrajdic D., Wymond B., Benassi-Evans B. (2022). Assessing the Effect of Plant-Based Mince on Fullness and Post-Prandial Satiety in Healthy Male Subjects. Nutrients.

[31] Pliner P., Hobden K. (1992). Development of a Scale to Measure the Trait of Food Neophobia in Humans. Appetite.

[32] Steptoe A., Pollard T.M., Wardle J. (1995). Development of a Measure of the Motives Underlying the Selection of Food: The Food Choice Questionnaire. Appetite.

[33] Lindeman M., Väänänen M. (2000). Measurement of Ethical Food Choice Motives. Appetite.

[34] Prochaska J.O., Velicer W.F. (1997). The Transtheoretical Model of Health Behavior Change. Am. J. Health Promot..

[35] Graça J., Calheiros M.M., Oliveira A. (2015). Attached to Meat? (Un)Willingness and Intentions to Adopt a More Plant-Based Diet. Appetite.

[36] IBM Corp (2021). Released 2021. IBM SPSS Statistics for Windows Version 28.0.

[37] Clark M.J., Slavin J.L. (2013). The Effect of Fiber on Satiety and Food Intake: A Systematic Review. J. Am. Coll. Nutr..

[38] Wanders A.J., van den Borne J.J.G.C., de Graaf C., Hulshof T., Jonathan M.C., Kristensen M., Mars M., Schols H.A., Feskens E.J.M. (2011). Effects of Dietary Fibre on Subjective Appetite, Energy Intake and Body Weight: A Systematic Review of Randomized Controlled Trials. Obes. Rev..

[39] Van Dongen M.V., De Graaf C., Siebelink E., Kok F.J. (2009). Hidden Fat Facilitates Passive Overconsumption. J. Nutr..

[40] Blundell J.E., de Graaf K., Finlayson G., Halford J.C.G., Hetherington M., King N.A., Stubbs J. (2009). Measuring Food Intake, Hunger, Satiety and Satiation in the Laboratory. Handbook of Assessment Methods for Eating Behaviours and Weight-Related Problems.

[41] Stribiţcaia E., Evans C.E.L., Gibbons C., Blundell J., Sarkar A. (2020). Food Texture Influences on Satiety: Systematic Review and Meta-Analysis. Sci. Rep..

[42] de Castro J.M. (1994). Family and Friends Produce Greater Social Facilitation of Food Intake than Other Companions. Physiol. Behav..

[43] Berridge K.C., Robinson T.E. (2003). Parsing Reward. Trends Neurosci..

[44] Bellisle F., Lucas F., Amrani R., Le Magnen J. (1984). Deprivation, Paiatability and the Micro-Structure of Meals in Human Subjects. Appetite.

[45] Yeomans M.R. (1996). Palatability and the Micro-Structure of Feeding in Humans: The Appetizer Effect. Appetite.

[46] Van Bergen G., Neufingerl N., Meijboom S., De Rosa Spierings K., Zandstra E., Polet I. (2023). What’s Cooking, If Not Meat? Effects of Repeated Home-Use, Recipe Inspiration and Meal Context on Perception of Plant-Based Meat Analogues. Appetite.

[47] Eckl M.R., Biesbroek S., Van’T Veer P., Geleijnse J.M. (2021). Replacement of Meat with Non-Meat Protein Sources: A Review of the Drivers and Inhibitors in Developed Countries. Nutrients.

[48] Sijtsema S.J., Dagevos H., Nassar G., de Winter M.v.H., Snoek H.M. (2021). Capabilities and Opportunities of Flexitarians to Become Food Innovators for a Healthy Planet: Two Explorative Studies. Sustainability.

[49] Rogers P.J., Schutz H.G. (1992). Influence of Palatability on Subsequent Hunger and Food Intake: A Retrospective Replication. Appetite.

[50] Bobroff E.M., Kissileff H.R. (1986). Effects of Changes in Palatability on Food Intake and the Cumulative Food Intake Curve in Man. Appetite.

[51] Mattes M.Z., Vickers Z.M. (2018). Better-Liked Foods Can Produce More Satiety. Food Qual. Prefer..

[52] Zandstra E.H., De Graaf C., Mela D.J., Van Staveren W.A. (2000). Short- and Long-Term Effects of Changes in Pleasantness on Food Intake. Appetite.

[53] Hill A.J., Magson L.D., Blundell J.E. (1984). Hunger and Palatability: Tracking Ratings of Subjective Experience before, during and after the Consumption of Preferred and Less Preferred Food. Appetite.

[54] Holt S.H.A., Delargy H.J., Lawton C.L., Blundell J.E. (1999). The Effects of High-Carbohydrate vs High-Fat Breakfasts on Feelings of Fullness and Alertness, and Subsequent Food Intake. Int. J. Food Sci. Nutr..

[55] Yeomans M.R., Symes T. (1999). Individual Differences in the Use of Pleasantness and Palatability Ratings. Appetite.

[56] Pater L., Kollen C., Damen F.W.M., Zandstra E.H., Fogliano V., Steenbekkers B.L.P.A. (2022). The Perception of 8- to 10-Year-Old Dutch Children towards Plant-Based Meat Analogues. Appetite.

[57] Zandstra E.H., Mathey M.F.A.M., De Graaf C., Van Staveren W.A. (2000). Short-Term Regulation of Food Intake in Children, Young Adults and the Elderly. Eur. J. Clin. Nutr..

[58] De Wijk R.A., Kaneko D., Dijksterhuis G.B., van Zoggel M., Schiona I., Visalli M., Zandstra E.H. (2019). Food Perception and Emotion Measured over Time In-Lab and in-Home. Food Qual. Prefer..

[59] Zandstra E.H., Lion R. (2019). In-Home Testing. Context: The Effects of Environment on Product Design and Evaluation.

[60] Lasschuijt M.P., de Graaf K., Mars M. (2021). Effects of Oro-Sensory Exposure on Satiation and Underlying Neurophysiological Mechanisms—What Do We Know so Far?. Nutrients.

[61] Meiselman H.L. (2013). The Future in Sensory/Consumer Research: Evolving to a Better Science. Food Qual. Prefer..

[62] Zandstra E.H., Stubenitsky K., De Graaf C., Mela D.J. (2002). Effects of Learned Flavour Cues on Short-Term Regulation of Food Intake in a Realistic Setting. Physiol. Behav..

